# Serglycin proteoglycans limit enteropathy in *Trichinella spiralis*-infected mice

**DOI:** 10.1186/s12865-016-0155-y

**Published:** 2016-06-08

**Authors:** Ananya Roy, Osama Sawesi, Ulrika Pettersson, Anders Dagälv, Lena Kjellén, Anna Lundén, Magnus Åbrink

**Affiliations:** Department of Medical Biochemistry and Microbiology, Uppsala University, Uppsala, Sweden; Department of Pathology and Wildlife Diseases, The National Veterinary Institute, Uppsala, Sweden; Department of Microbiology, The National Veterinary Institute, Uppsala, Sweden; Department of Biomedical Sciences and Veterinary Public Health, Section of Immunology, Swedish University of Agricultural Sciences, VHC, Box 7028, 75007 Uppsala, Sweden

**Keywords:** Mast cell, Parasite, Serglycin, Infection, *Trichinella spiralis*, Intestine

## Abstract

**Background:**

Serglycin proteoglycans are essential for maturation of secretory granules and for the correct granular storage of cationic proteases in hematopoietic cells, e.g. mast cells. However, little is known about the in vivo functions of serglycin proteoglycans during infection. Here we investigated the potential role of serglycin proteoglycans in host defense after infection with the nematode *Trichinella spiralis.*

**Results:**

Twelve days post infection lack of serglycin proteoglycans caused significantly increased enteropathy. The serglycin-deficient mice showed significantly increased intestinal worm burden, reduced recruitment of mast cells to the intestinal crypts, decreased levels of the mast cell proteases MCPT5 and MCPT6 in intestinal tissue, decreased serum levels of TNF-α, IL-1β, IL-10 and IL-13, increased levels of IL-4 and total IgE in serum, and increased intestinal levels of the neutrophil markers myeloperoxidase and elastase, as compared to wild type mice. At five weeks post infection, increased larvae burden and inflammation were seen in the muscle tissue of the serglycin-deficient mice.

**Conclusions:**

Our results demonstrate that the serglycin-deficient mice were more susceptible to *T. spiralis* infection and displayed an unbalanced immune response compared to wild type mice. These findings point to an essential regulatory role of serglycin proteoglycans in immunity.

**Electronic supplementary material:**

The online version of this article (doi:10.1186/s12865-016-0155-y) contains supplementary material, which is available to authorized users.

## Background

Serglycin is a proteoglycan mainly found in secretory granules, expressed by several hematopoietic cell types [1]. In these cells, e.g. in cytotoxic T cells, natural killer (NK) cells, neutrophils, platelets and mast cells (MCs) the serglycin proteoglycans contribute to granular integrity [2], and in the serglycin-deficient (SG^−/−^) mouse strain the storage/retention of inflammatory mediators, i.e. cationic proteins, is impaired. The cationic proteins affected by serglycin deletion include the connective tissue specific MC-proteases; the two chymases mouse mast cell protease 4 (MCPT4) and MCPT5, the tryptase MCPT6 (also designated mMCP-4, mMCP-5, mMCP-6, respectively), and carboxypeptidase A3 (CPA3). Other cationic proteins that are affected are the neutrophil elastase (NE), granzyme B, and platelet factor 4 [2]. The only serglycin-independent MC-specific protease identified so far is the mucosal MC-specific chymase MCPT1 (mMCP-1) [3]. In murine mucosal MCs, chondroitin sulfate type glycosaminoglycan chains are attached to serglycin [3], whereas serglycin in connective tissue MCs carries heparin [4, 5]. The type of glycosaminoglycan is important for correct storage of most connective tissue MC-specific mediators. When MCs degranulate, serglycin proteoglycan, proteases and other mediators are released to the surrounding tissue where they affect inflammatory and healing processes, and contribute to tissue homeostasis [6–8]. Under these conditions serglycin proteoglycans may serve as an important co-factor for the proteases and play a vital role in cytokine signaling [9].

Although several studies in vitro clearly show an important function of serglycin proteoglycans at the cellular level, e.g. in MCs, only a few studies have so far addressed the function of serglycin proteoglycans in vivo*,* where several cell-types involved in innate and acquired immune responses express serglycin proteoglycans. Host immune responses to the parasitic nematode *Trichinella spiralis* have been extensively studied, and MCs have been shown to contribute to worm expulsion 10 to 14 days post infection (dpi) and in the mounting of an efficient immune response [10–12]. MCPT1 is important for expulsion of adult worms after primary as well as secondary infection [13], and plays a role in the development of the parasite induced enteropathy in mice [14]. Furthermore, in chronically infected mice the connective tissue MC-specific tryptase MCPT6 is important for the recruitment of eosinophilic granulocytes to the infected muscle tissue, thereby contributing to the IgE-mediated killing of larvae [15].

To study the functional role of serglycin proteoglycans during parasite infection we infected mice with the nematode *Trichinella spiralis*. Expulsion of the adult worms from the gut was observed 12 dpi, and encapsulated larvae in the skeletal muscle cells were found 5 weeks post infection. Our results suggest that during infection with *T. spiralis,* serglycin proteoglycans play a pivotal role in both early and late host immune responses. Interestingly, we found that the lack of serglycin proteoglycans aggravates the enteropathy and cause dysregulated Type 2 cytokine responses at 12 dpi, leading to increased numbers of encysted larvae in skeletal muscle at 5 weeks post infection.

## Methods

### Animals and ethics statement

Generation of the SG^−/−^ mouse strain has been described previously [16]. The N12 generation, back-crossed to the C57Bl/6 J strain (from Taconic), was used to generate the SG^−/−^ and wild type (WT) C56BL/6 mouse lines. To evaluate whether the altered responses of the SG^−/−^ mice were due to an impared function of MCs or of other serglycin-containing cells, 7 SG^−/−^ mice were reconstituted intraperitoneally with 5 x10^6^ bone marrow derived SG^+/+^ MCs. Reconstituted animals (RSG^−/−^) were used in infection experiments 8 weeks after reconstitution. To evaluate the contribution of connective tissue type MCs and in particular the heparin-dependent proteases MCPT4, MCPT5, MCPT6 and CPA3, the *N*-deacetylase/N-sulfotransferase 2-deficient (NDST2^−/−^) mouse strain [4], which lacks heparin, was also used in infection experiments. Animals were allowed food and water ad libitum.

These studies were conducted at the National Veterinary Institute in Uppsala, Sweden and were carried out in full compliance with the guidelines of the Swedish Animal Welfare Agency. The regional ethical committee (Uppsala District Court) approved (permission C221/7 and C297/10) all of the animal studies included in this article. Care was taken to minimize animal suffering during handling and experimentation.

### Infection protocols

*Trichinella spiralis* (strain ISS03, Istituto Superiore di Sanita, Rome, Italy) was maintained in BALB/c mice and larvae were recovered by pepsin-acid digestion. In a series of experiments 8–10 week old WT (*n* = 4-7) and SG^−/−^ mice (*n* = 4-7) were inoculated by oral gavage with 500 *T. spiralis* larvae suspended in PBS with 0.1 % agar. Infected and non-infected control mice were killed at 12 days or 5 weeks post infection. For the various parameters measured results from up to 3 separate experiments were pooled. In the mast cell reconstitution experiment described above we infected 12–16 week old WT (*n* = 5), SG^−/−^ (*n* = 4), RSG^−/−^ (*n* = 4), and NDST2^−/−^ (*n* = 5) mice, with additionally three mice of each genotype used as uninfected controls, and analyzed the mice 12 dpi.

### Parasite burden and muscle pathology

Adult worm and larval burden were examined 12 days and 5 weeks post infection, respectively. Adult worms were recovered from the small intestine [17] after removal of the proximal 10 cm of the small intestine for histological examination and enzyme analyses. At 5 weeks, the diaphragm and masseter muscle were excised for histology and then the remaining whole carcasses were individually digested and the larvae counted. The masseter muscle from one cheek of each infected mouse was fixed in 4 % paraformaldehyde, paraffin embedded and processed using standard histological techniques and stained with Giemsa. Areas of inflammation around encapsulated *T. spiralis* were identified in a Nikon 90i microscope and areas of infiltrating leukocytes measured using Nikon NIS software. For each muscle section, 10 random areas with inflammatory cells were measured per infected mouse.

### Quantification of intestinal pathology

Intestinal architecture was assessed in the small intestine. See Additional file 1 for details. Briefly, a sample with the length of 10 cm next to the pylorus was excised and the distal ≤3 cm used for histopathology evaluation for both the control and infected animals. All histopathological parameters described, i.e. villus lengths, tip swelling, and epithelial lesions were done on tissue sections stained with haematoxylin and eosin (H&E) and measured using Nikon NIS software. Each villus length was measured from the tip of the villus to the junction with the crypt region. Villus tip swelling was measured as the breadth of the villus tip. A total of 15 villi and villi tips in one intestinal section per mouse were measured. Epithelial lesions were recorded as the number of vacuolized enterocytes along the villi tip lining (and as percentage of the enterocytes, data not shown), and lesions were counted in 10 villi per infected mouse.

### Detection of mast cells and other inflammatory cells

MCs in the intestine were detected by immunohistochemistry using antibodies towards CD117/c-kit (Abcam) and with Naphthol AS-D chloroacetate esterase (Sigma) staining. Macrophages were quantified using mouse F4/80 antibody. Granulocytes, neutrophils and eosinophils were counted in H&E stained intestine sections. See Additional file 1 for details.

### Western blot analysis of leukocyte proteases

Tissue from the small intestine (80–100 mg) was homogenized in PBS/0.1 mM EDTA/2 % Triton X-100 containing 2 M NaCl and the supernatants were used for analysis of MCPT5, MCPT6, NE, myeloblastin/proteinase 3 (PR3), eosinophil major basic protein (EMBP) and β-actin. Western blots were performed using a monoclonal antibody to β-actin (Santa Cruz Biotechnology), or polyclonal rabbit antiserum to EMBP (Santa Cruz Biotechnology), polyclonal goat antiserum to NE (Santa Cruz Biotechnology), polyclonal rabbit antiserum to PR3 (Santa Cruz Biotechnology), MCPT5 and MCPT6 (a kind gift from Lars Hellman, Uppsala University and Gunnar Pejler, Swedish University of Agricultural Sciences). Peritoneal cell-derived MCs were used as a positive control for detection of MCPT5 and MCPT6. Quantification of the protein bands was done with ImageJ software where relative intensity was measured in terms of intensity increase compared to the background. For details see Additional file 1.

### Measurement of MCPT1, cytokine levels and antibody responses

ELISA kits were used to quantify the concentration of MCPT1 in homogenates of intestinal tissue (e-Biosciences) and the concentration of TNF-α, IL-1β, IL-10, IL-4 and IL-13 in serum samples from uninfected and infected mice (PeproTech). Total serum IgE levels were measured by capture ELISA with an anti-mouse IgE IgG_1_ antibody (Southern Biotech, USA) used as capture antibody and alkaline phosphate conjugated anti-mouse IgE IgG_1_ as detection antibody (Southern Biotech, USA). A monoclonal IgE antibody (a kind gift from Jenny Hallgren, Uppsala University) was used as a standard to quantify IgE. To measure parasite specific IgG, *T. spiralis* larval homogenate was used as coating antigen at 10 μg/ml in the ELISA. IgG was detected using horseradish peroxidase-conjugated anti-mouse IgG. Enzyme activity was detected by addition of the substrate 2,2′-Azinobis [3-ethylbenzothiazoline-6-sulfonic acid]-di-ammonium salt (Sigma) and the absorbance measured at 405 nm.

### Myeloperoxidase and neutrophil elastase assay

MPO and NE activities were measured in snap frozen samples of the small intestine (80 to 100 mg). MPO was assesed biochemically by homogenizing the tissue in 400 μl of cold 1 % hexadecyl trimethyl ammonium bromide (Sigma) solution and then the supernatant was mixed with the substrates o-phenylenediamine to measure the enzymatic reaction at 490 nm. For NE activity the tissue was homogenised in Hank’s balanced salt solution and enzymatic activity measured at 405 nm using substrate Suc-Ala-Ala-Pro-Val-pNA(L-1770 BACHEM). See Additional file 1 for details.

### Statistical analysis

Statistical analyses were performed using GraphPad Prism 4.0 (GraphPad Software, San Diego, CA). Mann–Whitney *U* test was used for analysis of intestinal and muscle parasite burden. In all other instances statistical differences between groups were evaluated using Student’s *t* test (unpaired, two tailed), with *P*-values ≤0.05 considered significant. Results are presented as mean values, unless otherwise stated in the figure legends, with *P*-values indicated: not significant, ns *P* >0.05, *P ≤0.05, **P <0.001, ***P <0.0001 versus infected WT mice, or (ns) *P* >0.05, +*P* ≤0.05, ++*P* <0.001, +++*P* <0.0001 versus infected SG^−/−^ mice, and #*P* ≤0.05, ##*P* <0.001, ###*P* <0.0001 versus infected animals.

## Results

### Increased worm burden and enteropathy in *T. spiralis* infected serglycin-deficient mice

**Fig. 1:**
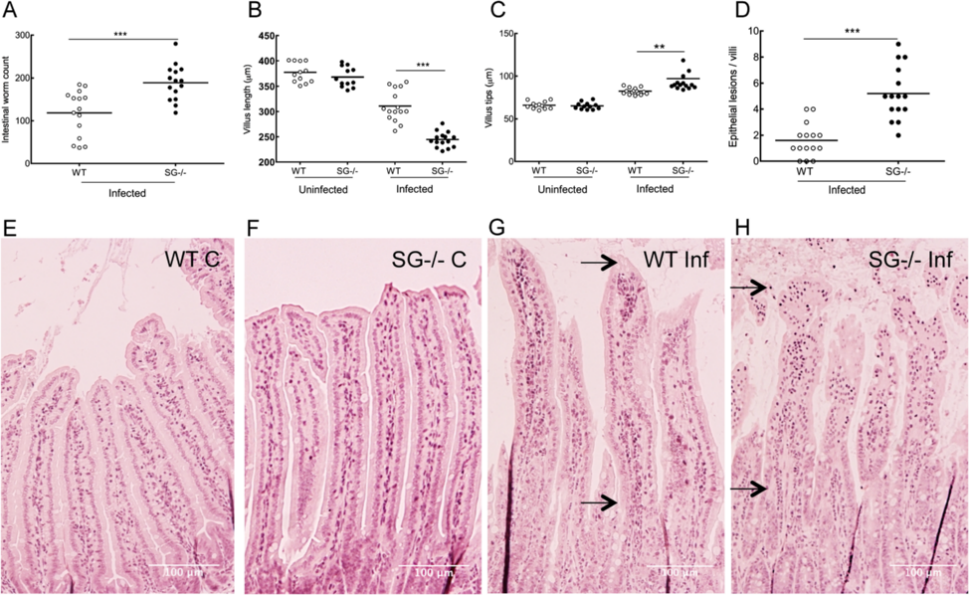
Serglycin proteoglycans limits *T. spiralis* induced entheropathy. **a** Intestinal worm burden. **b**-**h** Paraffin embedded intestinal tissues from control and infected WT and SG^−/−^ mice (12 dpi) were sectioned, stained with H&E and the histopathological changes observed. **b** Shortening of the villi and (**c**) swelling of the villi tips were quantified by measuring the length and breadth of 15 villi per intestinal section and mouse, using the Nikon NIS software. In (**d**) the epithelial lesions (as defined in M&M) were counted in 10 villi per infected mouse. Representative photographs of intestinal tissues of WT and SG^−/−^ mice used for the scoring presented in (**b**, **c**, **d**) are shown in (**e**-**h**). (**e**) Uninfected WT, (**f**) uninfected SG^−/−^, (**g**) infected WT and (**h**) infected SG^−/−^ sections of intestines at 100 X magnification. Arrows (in **g** and **h**) indicate the villi region where the length was measured. Pooled data from 3 experiments for each genotype is shown with control mice (*N* = 12) and infected mice (*N* = 15). Data is expressed as mean values (**a**-**d**) and significant differences between WT and SG^−/−^ mice are indicated in the figure. **P <0.001, ***P <0.0001 versus infected WT mice

To study the role of serglycin proteoglycans in an experimental *T. spiralis* infection model, WT and SG^−/−^ mice were inoculated with 500 infective muscle larvae by gavage. At 12 dpi the intestinal worm burden was significantly increased in SG^−/−^ mice compared to WT mice indicating that serglycin proteoglycans are involved in the expulsion of *T. spiralis* (Fig. 1a). Gastrointestinal nematode infections usually cause enteropathy, which can be measured as villus atrophy [18] and other pathological changes in the intestine [19, 20]. At 12 dpi, marked histopathological intestinal alterations were evident in both SG^−/−^ and WT mice and characterized by reduced villi length, swelling of the villi tips, more epithelial lesions of the villi and goblet cell hyperplasia as compared to non-infected control animals (Fig. 1b-h). While goblet cell hyperplasia (Additional file 2: Figure S1a) and *T. spiralis*-specific IgG levels in serum (Additional file 2: Figure S1b) were similar in infected SG^−/−^ mice and WT mice, infected SG^−/−^ mice displayed a significant reduction of villi length (Fig. 1b) and increased villi tip swelling (Fig. 1c) as well as more epithelial lesions of the villi (Fig. 1d and data not shown) compared to infected WT mice. This suggests that lack of serglycin proteoglycans lead to increased susceptibility to *T. spiralis* infection either by increased establishment or delayed expulsion of the worms. Hence, serglycin proteoglycans contribute to the control of the enteropathy, possibly through mechanisms mediated via its negatively charged glycosaminoglycan chains, i.e. as a co-factor of inflammatory mediators.

### *T. spiralis* infected serglycin-deficient mice express normal MCPT1 intestinal levels but has decreased intestinal mast cell recruitment and expression of MCPT5 and MCPT6

**Fig. 2:**
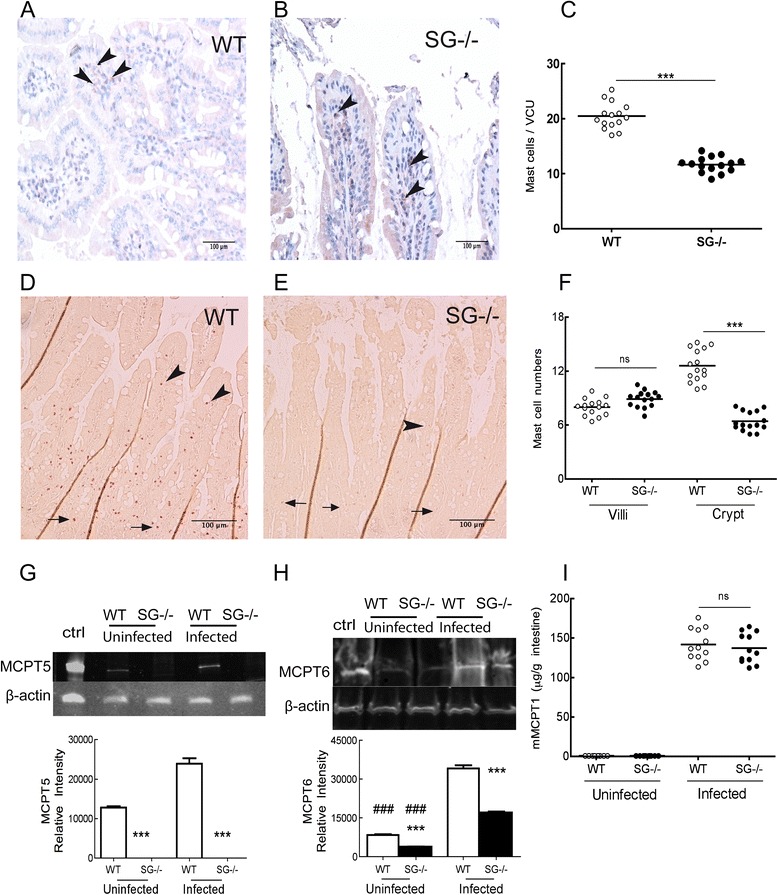
Serglycin proteoglycans contribute to mast cell recruitment and mast cell protease levels during *T. spiralis* induced enteropathy (12 dpi). Paraffin embedded intestines were sectioned and stained with CD117/c-kit antibody and Napthol AS-D chloroacetate esterase for detection and quantification of MCs. **a** WT and (**b**) SG^−/−^ intestinal sections at 200 X magnification stained with CD117; (**d**) WT and (**e**) SG^−/−^ sections at 100 X magnification stained with chloroacetate esterase. Arrows point to crypt MCs and arrowheads to villus MCs. **c** CD117 positive MC counts in 50 villus crypt units (VCUs) in infected WT and SG^−/−^ mice. In (**f**) counts of chloroacetate esterase stained MCs in 25 villus and 25 crypts in infected WT and SG^−/−^ mice are shown. In (**g**) intestinal levels of MCPT5 and (**h**) MCPT6 were analyzed by western blotting and the relative signal intensity estimated with Image J, and in (**i**) intestinal levels of MCPT1 was analyzed by ELISA. In **c** and **f**, pooled data from three experiments is shown with infected mice (*N* = 15) for each genotype. In **g** and **h**, the relative signal intensity is calculated on *N* = 6 for each genotype. In I, pooled data from 2 experiments is shown with infected mice (*N* = 12) for each genotype. Data is expressed as mean values (in **c**, **f, i**) and mean values + SEM (in **g, h**), and significant differences between genotypes are indicated in the figure. Not significant, ns *P* >0.05, ***P <0.0001 versus WT mice and ###*P* <0.0001 versus infected mice

Since MCs have been implicated in the expulsion phase of *T. spiralis* [13, 14], and MCs are severely affected by the deletion of serglycin [3, 16, 21–23], we next assessed if MC-recruitment and MC protease levels were affected in the SG^−/−^ mice at 12 dpi. Immunohistochemistry staining with CD117/c-kit-antibody showed MC numbers per villus crypt units (VCU) to be significantly lower in infected SG^−/−^ mice than in infected WT mice (Fig. *2*a, b, c). Furthermore, the numbers of chloroacetate esterase stained MCs, *i.e.* chymase positive MCs, were similar in the villi, but significantly lower in the crypts of infected SG^−/−^ mice in comparison to WT mice (Fig. *2*d, e, f). This suggests that serglycin proteoglycans may contribute to the recruitment of crypt MCs, whereas the migration of MCs to the villi seems to be serglycin-independent at 12 dpi. Interestingly, SG^−/−^ mice reconstituted with bone marrow derived SG^+/+^ MCs (RSG^−/−^) also had fewer MCs in the crypts (see Additional file 1: text and Additional file 3: Figure S2c).

In a recent study we showed that SG^−/−^ mice infected with *Toxoplasma gondii* had similar intraperitoneal levels of the two serglycin/heparin-dependent proteases MCPT5 and MCPT6 [16, 21], as infected WT mice [24]. In the present model, the intestinal MCPT5 levels increased in infected WT mice but remained undetectable in SG^−/−^ mice (Fig. 2g). In naïve and infected SG^−/−^ mice the MCPT6 levels were lower than in WT mice, but infected mice of both genotypes showed increased MCPT6 levels compared to non-infected control animals (Fig. 2h). In contrast, the mucosal chymase MCPT1, implicated in the expulsion of *T. spiralis* [13], was not affected by the lack of serglycin proteoglycans (Fig. 2i), suggesting that MCPT1 remains serglycin-independent during infection with *T. spiralis.* These findings on MC protease expression may reflect a difference in the immune response between tissues as well as in the response to different infectious agents.

### Decreased inflammatory cytokine levels in *T. spiralis* infected serglycin-deficient mice

**Fig. 3:**
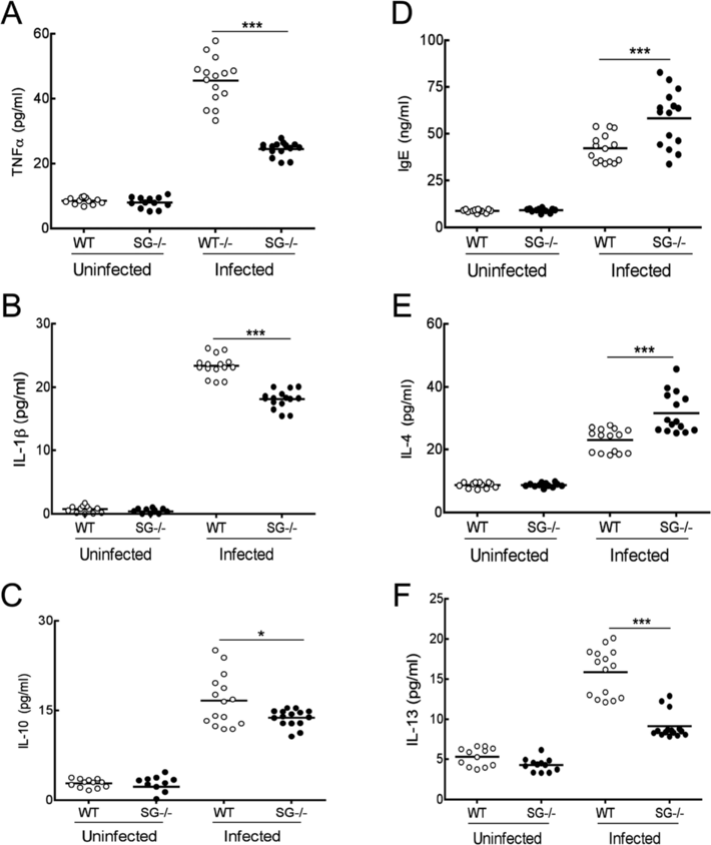
Serglycin proteoglycans enhance serum cytokine responses and affect IgE antibody levels in *T. spiralis* infected mice (12 dpi). The serum levels of TNF-α (**a**), IL-1β (**b**), IL-10 (**c**), total IgE (**d**), IL-4 (**e**) and IL-13 (**f**) were analyzed by ELISA. Pooled data from three experiments is shown with control mice (*N* = 12) and infected mice (*N* = 15) for each genotype. Data is expressed as mean values and significant differences between genotypes are indicated in the figure. *P ≤0.05, **P <0.001, ***P <0.0001 versus infected WT mice

Infection with *T. spiralis* induces increased systemic levels of pro-inflammatory cytokines [25, 26]. Since MCs and serglycin proteoglycans may contribute to the pro-inflammatory cytokine signaling during infection [2, 27], we analyzed the serum levels of the pro-inflammatory cytokines TNF-α and IL-1β, and the regulatory cytokine IL-10. The infection induced increased expression of TNF-α (Fig. 3a), IL-1β (Fig. 3b) and IL-10 (Fig. 3c) in both WT and SG^−/−^ mice, but the cytokine levels were significantly lower in the SG^−/−^ mice. Next we analyzed if reconstitution of SG^−/−^ mice with WT MCs, or the absence of heparin would affect the cytokine profiles. After *T. spiralis* infection RSG^−/−^ mice had significantly increased TNF-α and IL-1β levels compared to SG^−/−^ mice, although not as high as WT mice. Infected NDST2^−/−^ mice expressed significantly lower TNF-α levels compared to WT mice, while IL-1β and IL-10 levels were similar as WT mice (Additional file 4: Figure S3a, b, c).

### Increased IgE levels in *T. spiralis* infected serglycin-deficient mice

Induction of a Type 2 cytokine response profile, characterized by the cytokines IL-4, IL-13 and increased IgE, may be crucial for expulsion of helminth parasites [28, 29]. IL-4 and IL-13 are involved in the antibody isotype switch to IgG1 or IgE in B-cells, and implicated in MC development as well as in increasing sensitivity to inflammatory mediators [30]. To investigate the potential role of serglycin proteoglycans in the formation of a Type 2 cytokine response profile during *T. spiralis* infection, we analyzed the serum levels of total IgE, IL-4 and IL-13 at 12 dpi. The IgE levels increased in *T. spiralis* infected animals and were significantly higher in SG^−/−^ mice compared to WT mice (Fig. 3d). Infected WT and SG^−/−^ mice had significantly increased levels of IL-4 and IL-13 compared to non-infected control animals. However, while infected SG^−/−^ mice showed a significant increase in IL-4 levels (Fig. 3e) compared to WT mice, the levels of IL-13 were significantly lower than in WT mice (Fig. 3f). In addition, MC reconstitution did not significantly alter the IgE and IL-4 levels in infected RSG^−/−^ mice, but significantly increased the IL-13 levels compared to the SG^−/−^ mice, whereas NDST2^−/−^ mice expressed similar IgE and Type 2 cytokine levels as WT mice (Additional file 4: Figure S3d, e, f).

These results suggest that the serum level of IL-1β and IL-13 during a *T. spiralis* infection may partly depend on MCs, and that the levels of TNF-α may actually depend on serglycin/heparin in this infection model. Furthermore, the increased enteropathy, the reduced inflammatory cytokine levels, the altered IL-4 and IL-13 levels, and increased IgE levels suggest that serglycin proteoglycans have a regulatory role in the immune response during *T. spiralis* infection. However, the immune responses in *T. spiralis*-infected SG^−/−^ mice seem to be largely independent of MC function, at least MC function mediated via serglycin proteoglycans. Thus, other mechanisms must cause the aggravation of the *T. spiralis*-induced enteropathy in SG^−/−^ mice.

### Increased myeloperoxidase and neutrophil elastase activities in *T. spiralis* infected serglycin-deficient mice

**Fig. 4:**
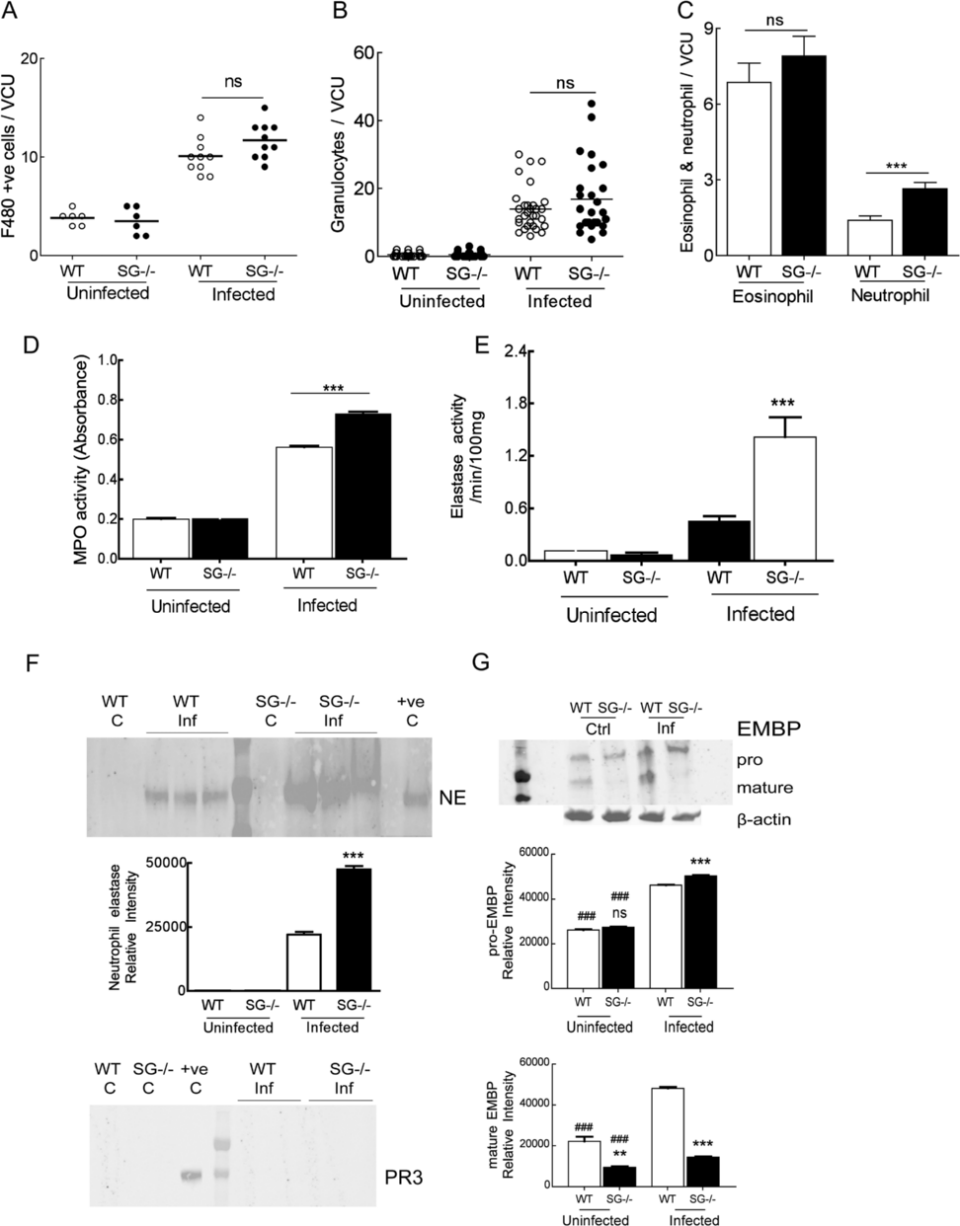
Serglycin proteoglycans influence neutrophil and eosinophil activity in *T. spiralis* infected mice (12 dpi). We assessed monocyte/macrophage (**a**) as well as neutrophil and eosinophil (**b**, **c**) recruitment in intestinal tissue in infected mice as compared with non-infected control mice, and measured the enzymatic activities of myeloperoxidase (MPO) (**d**) and neutrophil elastase (NE) (**e**) with substrate cleavage. We also quantified protein levels of NE and myeloblastin/proteinase 3 (PR3) (**f**) and eosinophil major basic protein (EMBP) (**g**) on western blots. In **a** data from two experiments are shown with control mice (*N* = 6) and with infected mice (*N* = 10). In **b**, **c**, **d** and **e** data from one representative experiment is shown with control mice (*N* = 3) and with infected mice (*N* = 3-5). In **f** and **g** a representative western blot is shown and a graph where the relative signal intensity (estimated with ImageJ) was calculated on *N* = 3-5 for each infected genotype and *N* = 3 for uninfected controls. Data is expressed as mean + SEM and significant differences are indicated in the figure. Not significant, ns *P* >0,05, **P <0.001, ***P <0.0001 versus infected WT mice, ##*P* <0.001, ###*P* <0.0001 versus infected mice

Given that we did not see a large impact of MCs on the intestinal enteropathy we investigated which other immune cells were involved in the infection. Macrophage (Fig. 4a) and total granulocyte (Fig. 4b) counts in the intestine were not significantly different between infected WT and infected SG^−/−^ mice. Eosinophils and neutrophils are quickly recruited to the intestine during a parasitic infection. We did not find any difference in the eosinophil numbers in the intestine of infected WT and SG^−/−^ mice, suggesting that recruitment of eosinophils is not affected by the lack of serglycin proteoglycans (Fig. 4c). However, neutrophil counts were significantly increased in infected SG^−/−^ compared to WT mice (Fig. 4c). Previously we have shown that the deletion of serglycin affected the levels of NE in naïve mice, whereas MPO levels were unaffected [31], and that early neutrophil recruitment was delayed in the SG^−/−^ mice during infection with *T. gondii* [24, 32]. Since enhanced neutrophil recruitment in the SG^−/−^ mice was observed, we next assessed neutrophil activity by measurements of MPO and NE activities in the intestinal tissue at 12 dpi. Infected SG^−/−^ mice showed significantly increased MPO and NE activity compared to infected WT mice (Fig. 4d, 4e). The MPO and NE activities were not dampened to WT levels in the MC reconstituted RSG^−/−^ mice, and NDST2^−/−^ mice showed similar MPO and NE activities as WT mice (Additional file 5: Figure S4a, b). Since the substrate used to determine the activity of NE can also be a substrate for myeloblastin/PR3 we used western blot to detect these two proteins in intestinal tissues. Infected SG^−/−^ mice had a significantly increased NE expression compared to WT mice, whereas myeloblastin/PR3 expression was undetectable at 12 dpi (Fig. 4f). These results suggest that serglycin proteoglycans influence neutrophil recruitment and the activities of NE and MPO in the intestinal tissue.

### Impaired activation of eosinophil major basic protein in serglycin-deficient mice

To assess the biological activity of eosinophils in the intestines the levels of EMBP were studied. EMBP is a potent cytotoxin/helminth-toxin that can interact with heparan sulfate glycosaminoglycans [32, 33]. The levels of the proform of EMBP in uninfected SG^−/−^ mice were similar to the levels found in naïve WT mice. Upon infection the levels of the proform increased and infected SG^−/−^ mice had a small but significant increase of the proform compared to WT mice (Fig. 4g). However, the amount of mature EMBP in intestinal tissue was significantly lower in SG^−/−^ mice than in WT mice, in uninfected as well as infected mice (Fig. 4g), indicating that serglycin proteoglycans may be involved in the activation of EMBP. This finding suggests that, unless EMBP need to be processed in a serglycin-dependent manner in the extracellular matrix after release, serglycin proteoglycans are expressed by eosinophils.

### Increased larval burden and inflammation in muscle tissue of *T. spiralis* infected serglycin-deficient mice

**Fig. 5:**
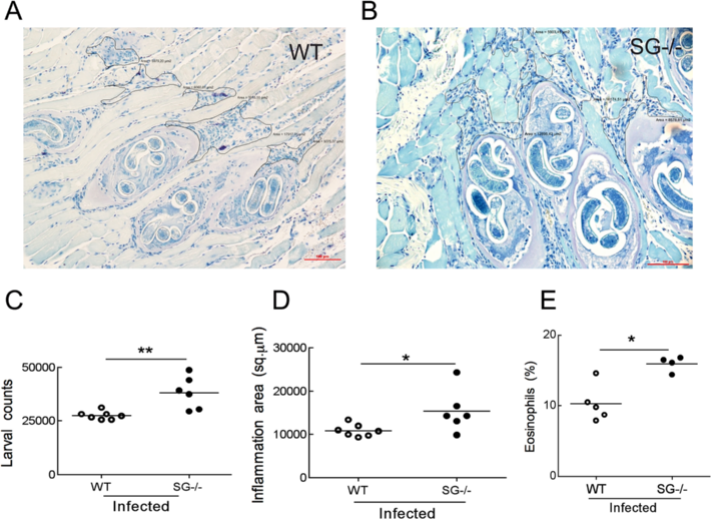
Serglycin-deficiency leads to increased muscle tissue larvae burden in *T. spiralis* infected mice. Larvae burden 5 weeks post infection was assesed in infected WT and SG^−/−^ mice muscle tissue stained with Giemsa. Representative pictures of cheek masseter muscle from SG^−/−^ (**a**) and WT (**b**) mice stained with Giemsa, with quantification of total larvae burden (**c**), area of inflammatory foci (**d**) and eosiniphil number in the inflammatory foci (**e**) are shown. Area of inflammation was determined with Nikon NIS software. Data obtained from one experiment with control mice (*N* = 3) and infected mice (*N* = 7 for WT and *N* = 6 for SG^−/−^) for each genotype is shown in **c** and **d**. *N* = 5 for WT and *N* = 4 for SG^−/−^ in **e**. Data is expressed as mean values and significant differences between WT and SG^−/−^ mice are indicated in the figure. *P ≤0.05, **P <0.001 versus infected WT mice

Seeing the prominent effects at 12 dpi in the SG^−/−^ mice, we finally evaluated if serglycin proteoglycans also play a role 5 weeks post infection in the chronic phase of *T. spiralis* infection. In the striated muscle tissue, the larvae invade myotubule cells and convert them into nurse cells that finally form an encysted larva/cell complex (Fig. 5a, b). At 5 weeks post infection, when counting larvae of whole carcasses, the SG^−/−^ mice harbored significantly more larvae than WT mice (Fig. 5c), and showed significantly increased areas of inflammation with increased eosinophil numbers around the larvae in the masseter muscle tissue compared to infected WT mice (Fig. 5d, 5e). This suggests that lack of serglycin proteoglycans continues to cause problems for the mice at 5 weeks post infection.

## Discussion

Sentinel cells, e.g. MCs, macrophages and dendritic cells contribute to the initial inflammatory reaction and the rapid recruitment of neutrophils and eosinophils to the site of infection. These cells also contribute significantly to initial development and modulation of the ensuing adaptive immune responses [34, 35]. Infection with *Trichinella spiralis* usually causes a strong polarization towards a Type 2 cytokine response [26, 36]. Expulsion of *T. spiralis,* which depends on several cooperative mechanisms [19, 20, 37, 38], normally occurs at day 10 to 14 in the mouse. In this study we aimed at investigating the functional role of serglycin proteoglycans in vivo during *T. spiralis* infection. We evaluated the histopathological changes in the intestinal mucosa, as well as the cellular responses, the pro-inflammatory signals and the cytokine profile. We included the NDST2^−/−^ mice, which lacks heparin and thus makes them a connective tissue MC deficient model.

At 12 dpi, the serglycin-deficient mice had significantly more worms in the gut and more pronounced morphological and inflammatory changes in the intestine than WT mice. Furthermore, the serglycin-deficient mice seemed to lack the proper control of the immune responses, with decreased levels of connective tissue type MC proteases, pro-inflammatory cytokines and altered Type 2 cytokine responses (Figs. 1, 2 and 3).

Ierna and colleagues have shown that soluble TNF-α is required for expulsion of *T. spiralis* in mice [27], and IL-1β has been suggested to be an important initiator of the inflammatory response during infection [25]. MC-derived TNF-α and IL-1β may also play an important role in inflammatory settings, where TNF-α can enhance T cell activation [39, 40], and IL-1β is important in the onset of arthritic inflammation [41]. In the serglycin-deficient mice, the levels of TNF-α and IL-1β were significantly lower, and the heparin-deficient NDST2^−/−^ mice had significantly less TNF-α, suggesting that heparin-expressing MCs also influence the levels of pro-inflammatory cytokines expressed during a *T. spiralis* infection. Furthermore, the significantly lower levels of TNF-α and IL-1β found in the *T. spiralis* infected serglycin-deficient mice also suggested that serglycin via its glycosaminoglycan chains may contribute to cytokine stability once secreted from MCs or macrophages. In addition, serglycin proteoglycans may contribute to activation or degradation of cytokines via serglycin-dependent MC-derived proteases. Interestingly, the IL-1β cytokine levels were increased by MC reconstitution, supporting the data showing that MCs play an important role in IL-1β production during inflammation [41].

Other studies have shown a critical role for MCs and the MC protease MCPT1 in *T. spiralis* infection [13, 42, 43]. When we assessed the intestinal tissue levels of the mucosal and connective tissue type MC proteases MCPT1, MCPT5 and MCPT6, the serglycin-deficient mice expressed significantly lower levels of the connective tissue type proteases, which correlated with decreased intestinal MC numbers. Interestingly, intestinal MCPT1 levels were not changed, supporting the in vitro data showing that MCPT1 indeed is serglycin-independent [3]. In contrast, the altered connective tissue MC protease levels suggest that they may confer some protection against the *T. spiralis* induced enteropathy. However, the intra-peritoneal reconstitution of the SG^−/−^ mice with serglycin-competent bone marrow derived MCs did not correct the aggravated phenotype, suggesting that the reduced levels of the serglycin-dependent connective tissue type MC proteases MCPT5 and MCPT6 play only a minor role in the aggravated enteropathy found in infected SG^−/−^ mice and that mucosal MCs play a more vital role. The finding that heparin-deficient NDST2^−/−^ mice, lacking connective tissue type MC proteases, mount a response equal to that of WT mice supports this notion.

Total IgE is increased as a result of the *T. spiralis* infection and the response in serglycin-deficient animals was significantly stronger than in WT animals, suggesting that serglycin proteoglycans may be involved in the control of cytokine levels, such as IL-4 and IL-13, which can induce the Type 2 cytokine profile and B-cell switch to IgE-production. Surprisingly, at 12 dpi, infected SG^−/−^ mice showed an inverse relation of the levels of IL-4 and IL-13, with significantly increased IL-4 but decreased IL-13 levels. Both IL-4 and IL-13 are important in host defense against *T. spiralis* [29, 44]*,* likely with different regulatory pathways [45], where NK cell derived IL-13 may cause some of the pathology associated with infection [46]. Furthermore, *T. spiralis* infection in IL-13^−/−^ mice resulted in significantly reduced intestinal pathology [29]. In contrast, the SG^−/−^ mice display aggravated enteropathy despite low levels of IL-13 suggesting that other serglycin-dependent mediators overrule the effects of lowered IL-13 levels. Interestingly, the reconstitution with serglycin-competent bone marrow derived MCs restored the IL-13 levels almost to WT levels, suggesting that serglycin-competent MCs may contribute an important part of the IL-13 secreted during infection. Alternatively, serglycin-competent MCs may act indirectly to recruit other IL-13-secreting leukocytes. This supports the notion that MCs may regulate the systemic levels of IL-1β through serglycin-dependent IL-13 secretion [47].

Deletion of serglycin affects many proteins normally stored in secretory granula in different cell types (reviewed in [2]), and aging SG^−/−^ mice (>9 months) frequently display enlarged lymphoid tissues without signs of infectious agents suggesting a functional role for serglycin proteoglycans in homeostasis [48]. In this study we have shown that despite the low levels of pro-inflammatory cytokines during a *T. spiralis* infection, the SG^−/−^ mice respond with increased intestinal erosion, which led us to investigate the overall levels of neutrophil and eosinophil derived cytotoxins/helminth-toxins. The level of MPO activity, a commonly used indicator of neutrophil infiltration and accumulation in inflammatory tissues [14], as well as neutrophil number was found to be significantly increased in the SG^−/−^ mice as compared to WT mice. We also found greatly increased levels of NE in the SG^−/−^ mice after *T. spiralis* infection (Fig. 4), much higher than the levels normally found in naive SG^−/−^ mice [31], suggesting that serglycin proteoglycans play an important regulatory role in neutrophil recruitment and in the secretion of NE from activated neutrophils. The increased levels of the cytotoxins MPO and NE may partly explain the increased villi erosion found in SG^−/−^ mice, but other cytotoxins could also be involved.

EMBPs have been shown to be potent killers of *T. spiralis* new born larvae [49, 50]. Interestingly, although the levels of proEMBP as well as the eosinophil numbers in infected WT and SG^−/−^ mice were similarly increased, the SG^−/−^ mice seem incapable of processing EMBP into its active form (Fig. 4). This is the first report suggesting a role for serglycin proteoglycans in the processing of EMBP, an observation that requires further studies. Furthermore, the lack of active EMBP may also contribute to the increased numbers of encysted larvae in muscle tissue at 5 weeks in the SG^−/−^ mice (Fig. 5).

In summary, we show that the SG^−/−^ mice respond by enhanced enteropathy to the *T. spiralis* infection, suggesting an important role of serglycin proteoglycans in the mounting of mucosal immune responses during infection with *T. spiralis*. The changed levels of IL-4 and IL-13 together with the altered pro-inflammatory cytokine profiles, the increased levels of IgE, impaired protease levels, and the aggravated enteropathy suggest that the balance between the Th1 and Th2 response profiles may be corrupted in the SG^−/−^ mice, a notion that requires further investigation. How serglycin proteoglycans act as co-factor in the regulation of the levels of cytokines and proteases remains elusive but the negatively charged glycosaminoglycan chains attached to serglycin may offer an interactive surface for regulatory cytokines. During infection in the SG^−/−^ mice, many of the proteins that depend on serglycin proteoglycans for storage in granular cells of uninfected mice, appear at the site of infection (at normal or even elevated levels), further strengthening and supporting the suggestion that serglycin proteoglycans are mainly involved in the correct storage of cationic proteins in granulated cells. The changed levels of inflammatory mediators indicate that serglycin proteoglycans also may have a regulatory role to play in the expression levels of these proteins. Importantly, our data suggests that during infection and inflammation, leukocytes may switch from a serglycin-dependent storage mode into a serglycin-independent mode of constitutive expression and secretion, as seen for cytotoxic T cells expressing granzyme B during virus infection [51], for MCs expressing the MC-specific proteases MCPT4, MCPT5, MCPT6 and CPA3 during infection with *T. gondii* [24], and for neutrophils expressing elastase (NE) during *T. spiralis* infection as shown in this study.

## Conclusion

Here we have shown that deletion of the serglycin core protein affects host immune responses to *T. spiralis* and results in increased susceptibility with higher parasite burden and aggravated enteropathy. Serglycin proteoglycan is highly expressed by several immune cells and the targeted deletion of serglycin has been shown to affect granule formation in mast cells, neutrophils, NK-cells and cytotoxic T cells. Thus, it is likely that serglycin expression contributes to the regulation and efficiency of immune responses. However, serglycin is apparently not essential for surviving a *T. spiralis* infection.

## Abbreviations

CPA3, carboxypeptidase A3; Dpi, days post infection; EDTA, ethylenediaminetetraacetic acid; ELISA, enzyme-linked immunosorbent assay; EMBP, eosinophil major basic protein; H&E, hematoxilin & eosin; Ig, immunoglobulin; IL, interleukin; M&M, materials & methods; MC/MCs, mast cell(s); MCPT, mast cell protease; mMCP, mouse mast cell protease; MPO, myeloperoxidase; NDST-2^−/−^, *N*-deacetylase/N-sulfotransferase 2-deficient mice; NE, neutrophil elastase; NK, natural killer; PBS, phosphate buffered saline; PR3, proteinase 3; RSG^−/−^, serglycin-deficient mice reconstituted with wild type mast cells; SG^−/−^, serglycin-deficient; TNF, tumour necrosis factor; VCU, villus crypt units; WT, wild type.

## Additional files

Additional file 1: Supplementary material & methods and results. (DOCX 21 kb)

Additional file 2: Figure S1. T. spiralis infection in serglycin-deficient mice. Paraffin embedded intestinal tissues from control and infected WT and SG^−/−^ mice (12 dpi) were sectioned and stained with H&E and the histopathological changes were observed. In (a) goblet cell hyperplasia was evaluated. Serum was collected from infected WT (*N* = 15) and serglycin-deficient (*N* = 15) mice and in (b) T.spiralis specific IgG levels was measured. Not significant, ns *P* >0.05 (TIF 241 kb)

Additional file 3: Figure S2. T. spiralis induced entheropathy in serglycin-deficient mice reconstituted with bone marrow derived wild type MCs and in heparin-deficient NDST2^−/−^ mice (12 dpi). The SG^−/− ^mice were reconstituted with 5 x106 WT MCs administered intraperitoneally and 8 weeks later infected with T. spiralis. (a) a representative intestinal section of the infected RSG^−/−^ mice stained with Toluidine blue (pH < 2.0), and (b) a representative cytospin slide of peritoneal cells from an infected RSG^−/−^ mice stained with May-Grünwald/Giemsa. Arrowheads mark the stained MCs. Paraffin embedded intestinal tissues from uninfected and infected WT, SG^−/−^, MC-reconstituted SG^−/−^ (RSG^−/−^), and NDST2^−/−^ mice, were sectioned and stained with H&E, and histopathological changes analyzed using Nikon NIS software. (c) MC-counts in chloroacetate esterase stained intestinal tissue, (d) intestinal worm burden, and (e) T. spiralis specific IgG levels in serum. In (f) the villus length, and (g) the swelling of the villi tips was measured of 15 villi per intestinal section as described in M&M. In (h) the epithelial lesion of the villus tip (as defined in Material and Methods) was counted in 10 intact villi per infected mouse. Data from one experiment is shown, with infected mice (*N* = 4 for RSG^−/−^ and SG^−/−^, *N* = 5 for WT and NDST2^−/−^) and with control mice (*N* = 3). Data is expressed as mean values (c, d, f-h) and mean + SEM (e), and significant differences between genotypes are indicated in the figure. Not significant, ns *P* >0.05, *P ≤0.05, **P <0.001, versus infected WT, and not significant (ns) *P* >0.05, ++*P* <0.001 versus infected SG^−/−^ mice, respectively. (TIF 9451 kb)

Additional file 4: Figure S3. T. spiralis induced cytokine and Th2 profile in heparin-deficient NDST2^−/−^ mice and SG-deficient mice reconstituted with bone marrow derived WT MCs (12 dpi). The levels in serum of TNF-α (a), IL-1β (b), and IL-10 (c), total IgE (d), IL-4 (e), and IL-13 (f) from uninfected and infected WT, SG^−/−^, MC-reconstituted SG^−/−^ (RSG^−/−^), and NDST2^−/−^ mice were analyzed by ELISA. Data from one experiment is shown, with infected mice (*N* = 4 for RSG^−/−^ and SG^−/−^, or *N* = 5 for WT and NDST2^−/−^) and with control mice (*N* = 3). A statistical significant difference in the levels of (c) IL-10 (WT vs SG^−/−^, p-value = 0,1294) and (e) IL-4 (WT vs SG^−/−^, *p*-value = 0,0758) was not reached in this experiment due to the low number of animals (compare with Fig. 3c and 3e). Data is expressed as mean + SEM and significant differences are indicated in the figure. #*P* ≤0.05, ##*P* <0.001, ###*P* <0.0001 versus infected mice. Not significant, ns *P* >0.05, *P ≤0.05, **P <0.001, ***P <0.0001 versus infected WT mice, and not significant (ns) *P* >0.05, +*P* ≤0.05, ++ *P* <0.001 versus infected SG^−/−^ mice, respectively. (TIF 1162 kb)

Additional file 5: Figure S4. Reconstitution with WT bone marrow derived MCs in serglycin-deficient mice does not dampen the enhanced neutrophil activity (at 12 dpi). Serglycin proteoglycans seem to influence neutrophil and eosinophil activities in T. spiralis infected mice (see Fig. 4 in the main text), and to assess neutrophil and eosinophil recruitment in intestinal tissue in infected serglycin-deficient mice repaired with WT MCs, we measured the enzymatic activities of (a) myeloperoxidase (MPO) and (b) neutrophil elastase (NE). In a and b data from one reconstitution experiment is shown with control mice (*N* = 3) and with infected mice (*N* = 4 for RSG^−/−^ and SG^−/−^, *N* = 5 for WT and NDST2^−/−^). Data is expressed as mean + SEM and significant differences are indicated in the figure. ##*P* <0.001, ###*P* <0.0001 versus infected mice. Not significant, ns *P* >0.05, **P <0.001, ***P <0.0001 versus infected WT mice, and not significant (ns) *P* >0.05, +++*P* <0.0001 versus infected SG^−/−^ mice, respectively. (TIF 397 kb)
