# Supplementary material for: Serglycin proteoglycans limit enteropathy in Trichinella spiralis-infected mice
Source: BMC Immunol. 2016 Jun 8;17:15. doi: 10.1186/s12865-016-0155-y (PMC4897876; doi:10.1186/s12865-016-0155-y)
Supplement: Supplementary file 1 — Supplementary material & methods and results. (DOCX 21 kb) [file 12865_2016_155_MOESM1_ESM.docx]

**Supplement to**

**Serglycin proteoglycans limit enteropathy in Trichinella spiralis- infected mice**

**Ananya Roy^1,4^, Osama Sawesi^1,4^, Ulrika Pettersson^2^, Anders Dagälv^1^, Lena Kjellén^1^, Anna Lundén^3^, Magnus Åbrink^4*^**

**Supplementary Material and Methods**

*Intestinal pathology*

Intestinal architecture was assessed in the small intestine. In control and infected animals a sample with the length of 10 cm next to the pylorus was excised and the distal ≤ 3 cm used for histopathology evaluation. The proximal 7 cm was snap frozen and used for biochemical assays and western blots. Tissue samples were fixed in 4% paraformaldehyde, paraffin embedded and processed using standard histological techniques and stained with haematoxylin and eosin (H&E). In serial sections from each individual mouse the morphology of ≥30 the villi-crypt units (VCUs) were scored in a blinded fashion. When scoring the three parameters (villi length, villus tip swelling, and epitelial lesions) care was taken to choose areas of the intestines with intact VCUs and from at least three different sections from each animal to counteract eventual processing variations. All histopathological parameters described, i.e. villus lengths, tip swelling, and epithelial lesions were observed in a Nikon 90i microscope and measured using Nikon NIS software. Each villus length was measured from the tip of the villus to the junction with the crypt region. Villus tip swelling was measured as the breadth of the villus tip. A total of 15 villi and villi tips in one intestinal section per mouse were measured. Epithelial lesions were recorded as the number of vacuolized enterocytes along the villi tip lining per viewing field, and lesions were counted in 10 intact villi tips per infected mouse. Alternatively, we also counted the ratio (percentage) of vacuolized cells per enterocytes in the villi tips (data not shown). To detect goblet cells, intestinal sections were stained with periodic acid-Schiff stain and counter stained with H&E, and the numbers of goblet cells were counted in 10 villi per mouse.

*Detection of mast cells and other inflammatory cells*

MCs in the intestine were detected by immunohistochemistry using antibodies towards CD117 (Abcam) and with Naphthol AS-D chloroacetate esterase (Sigma) staining. The CD117-stained MCs were counted in 50 villus crypt units (VCUs) and the MC counts expressed as per VCUs. When using chloroacetate esterase, MCs in the villi and crypt zones were counted separately to take into consideration the distribution of MCs along the crypt-villus axis. Care was taken to count only cells with round nuclei to avoid counting neutrophils. The protocol was developed for a selective staining of mast cell esterase-activity in most tissues. The chloroacetate esterase staining of mast cells is highly chymase dependent (Waern et al. J Immunol 183(10):6369-76, 2009), and chymase is a mast cell specific protease. Importantly, the deletion of serglycin affects the storage of most mast cell proteases and the chymase is basically completely gone (Åbrink et al. JBC 279(39): 40897–905, 2004).

To control that reconstitution of the SG-/- mice was successful, May-Grünwald/Giemsa stained MCs were counted among peritoneal exudate cells, and toluidine blue (pH<2.0) (Sigma) stained MCs were counted in villi and crypts of the intestines. Mouse F4/80 antibody was used to identify and quantify macrophages in the intestine. Marcophages in both crypts and villi were taken into account. To assess recruitment of granulocytes, eosinophils and neutrophils in infected intestines slides were stained with H&E. Cells with a polymorphic nucleus were counted in a blinded fashion in at least 15 VCUs per infected mouse. Initially, all cells with a polymorphic nucleus were counted. Then cells with a polymorphic nucleus with or without eosin staining were counted. Polymorphonuclear cells without eosin stain were considered as neutrophils and cells with eosin staining as eosinophils.

*Western blot analysis of leukocyte proteases*

Tissue from the small intestine (80-100mg) was homogenized in PBS/0.1mM EDTA/2% Triton X-100 containing 2M NaCl and left on ice for 15 min. Following centrifugation at 13,000 rpm for 20 min at 4°C, the supernatants were used for analysis of MCPT5, MCPT6, neutrophil elastase (NE), myeloblastin/proteinase 3 (PR3), eosinophil major basic protein (EMBP) and β-actin. Western blots were performed using a monoclonal antibody to β-actin (Santa Cruz Biotechnology), or polyclonal rabbit antiserum to EMBP (Santa Cruz Biotechnology), polyclonal goat antiserum to NE (Santa Cruz Biotechnology), polyclonal rabbit antiserum to proteinase 3 (Santa Cruz Biotechnology), MCPT5 and MCPT6 (a kind gift from Lars Hellman, Uppsala University and Gunnar Pejler, Swedish University of Agricultural Sciences). Peritoneal cell-derived MCs (46) were used as a positive control for detection of MCPT5 and MCPT6. Briefly, samples were separated under reducing conditions on 4-12% gradient SDS-PAGE gels (BioRad). Proteins were blotted onto Immobilon-FL transfer membranes (Millipore), followed by blocking with Odyssey blocking buffer for 1h, and then incubated with the specific antibodies overnight in the same buffer containing 0.05% Tween 20. Following incubation, the membrane was washed and incubated for 45 minutes with a secondary antibody donkey anti-rabbit IRDye 800 CW (LI-COR Biosciences) for detection of MBP, MCPT5, MCPT6 and myeloblastin/PR3, or with a donkey anti-goat IRDye 680 CW (LI-COR Biosciences) for detection of NE (secondary antibodies were diluted 1/4000 in 0.05% Tween 20). Membranes were then scanned using the Odyssey scanner (LI-COR Biosciences) for visualizing the bands. For β-actin, the membrane was incubated with horseradish peroxidase (HRP)-conjugated polyclonal goat anti-mouse IgG and then visualized using ECL (GE Healthcare) developing system. Quantification of the protein bands was done with ImageJ software where relative intensity was measured in terms of intensity increase compared to the background.

*Measurement of MCPT1, cytokine levels and antibody responses*

An ELISA kit (e-Biosciences) was used to quantify the concentration of MCPT1 in homogenates of intestinal tissue. ELISA kits (PeproTech, EC) were also used to quantify the concentration of TNF-α, IL-1β, IL-10, IL-4 and IL-13 in serum samples from uninfected and infected mice (with triplicates of each sample and the ELISA repeated twice) according to the manufacturer’s instructions. Total serum IgE levels were measured by capture ELISA with an anti-mouse IgE IgG1 antibody (Southern Biotech, USA) used as capture antibody and alkaline phosphate conjugated anti- mouse IgE IgG1 as detection antibody (Southern Biotech, USA). A monoclonal IgE antibody specific for TNP (a kind gift from Jenny Hallgren, Uppsala University) was used as a standard to quantify IgE. To measure parasite specific IgG, T. spiralis larval homogenate was used as coating antigen at 10 μg/ml in the ELISA. IgG was detected using HRP-conjugated anti-mouse IgG. Enzyme activity was detected by addition of the substrate 2,2'-Azinobis [3-ethylbenzothiazoline-6-sulfonic acid]-di-ammonium salt (Sigma) and the absorbance measured at 405 nm.

*Myeloperoxidase and neutrophil elastase assay*

Myeloperoxidase (MPO) and neutrophil elastase (NE) activities were measured in snap frozen samples of the small intestine (80 to 100 mg). The tissue was homogenized in 400 μl of cold 1% hexadecyl trimethyl ammonium bromide (Sigma) solution in phosphate buffer (pH 6). This was followed by sonication for 20 seconds and three rounds of snap freezing and thawing. The homogenate was centrifuged for 15 minutes at 12,000g at 4°C, and the supernatant was used for MPO assays. Ten μl of the supernatant was mixed with 200 μl of 50 mmol/L phosphate buffer (pH 6), containing 0.4 mg/ml of substrate o-phenylenediamine and 0.05% H2O2. The reaction was stopped after 20 minutes by adding 50 μl of 0.4 mol/L H2SO4 and the absorbance at 490 nm was measured. To assay NE activity, the sections of frozen intestine were homogenized in 2ml of Hank’s balanced salt solution with a homogenizer. The homogenate was then sonicated using 5X 30s pulses, centrifuged at 15000g for 30 min at 4°C, and the supernatant used to assess the NE activity. Supernatants were incubated with the elastase substrate (L-1770 BACHEM) in a final volume of 200μl of reaction buffer (100mMTris-HCl, 150mM NaCl, 0.05% Tween-20 and 0.1% BSA, pH 8.5). Incubation was performed at room temperature and the absorbance was measured at 405 nm in intervals between 5 and 30 minutes up to a period of 24 hours.

**Supplementary results**

*Reconstitution with bone marrow derived wild type mast cells in serglycin-deficient mice and infection with* T. spiralis

Successful reconstitution of the SG^-/-^ mice was verified by the identification of normally stained MCs in the intestine (supplement Fig. 2a) and in peritoneal lavage (supplement Fig. 2b). The heparin-deficient NDST2^-/-^ mouse strain that display defective connective tissue type MCs but have normal mucosal type MCs was included to directly assess the potential contribution of heparin-expressing MCs to the *T. spiralis*-induced enteropathy. Intestinal tissue MC-counts indicated that reconstituted SG^-/-^ (RSG^-/-^) mice failed to recruit more crypt MCs than SG^-/-^ mice (supplement Fig. 2c). The RSG^-/-^ mice showed similar intestinal worm counts as SG^-/-^ mice, whereas NDST2^-/-^ mice had similar intestinal worm counts as WT mice (supplement Fig. 2d). Infected RSG^-/-^ and NDST2^-/-^ mice had similar levels of *T. spiralis*-specific IgG in serum as infected SG^-/-^ and WT mice (supplement Fig. 2e). Interestingly, the RSG^-/-^ mice showed a slight, but significant, recovery of villus length (supplement Fig. 2f), but no reduction of villus tip swelling (supplement Fig. 2g) and epithelial lesions (supplement Fig. 2h and data not shown) as compared to the SG^-/-^ mice, whereas the enteropathy in the NDST2^-/-^ mice was found to equal the level of enteropathy in the WT mice (supplement Fig. 2f, g, h).
