# Supplementary figures and images for: Serglycin proteoglycans limit enteropathy in Trichinella spiralis-infected mice
Source: BMC Immunol. 2016 Jun 8;17:15. doi: 10.1186/s12865-016-0155-y (PMC4897876; doi:10.1186/s12865-016-0155-y)

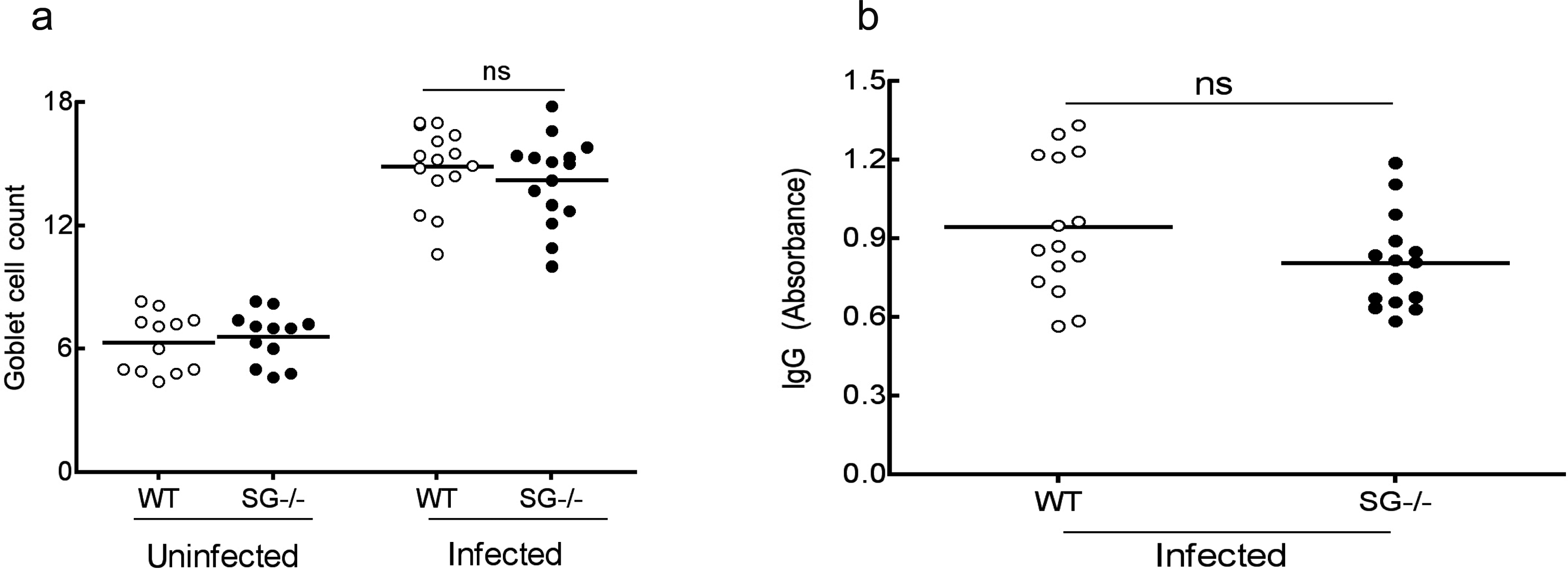

Supplement: Supplementary file 2 — T. spiralis infection in serglycin-deficient mice. Paraffin embedded intestinal tissues from control and infected WT and SG−/− mice (12 dpi) were sectioned and stained with H&E and the histopathological changes were observed. In (a) goblet cell hyperplasia was evaluated. Serum was collected from infected WT (N = 15) and serglycin-deficient (N = 15) mice and in (b) T.spiralis specific IgG levels was measured. Not significant, ns P >0.05 (TIF 241 kb) [file 12865_2016_155_MOESM2_ESM.tif]

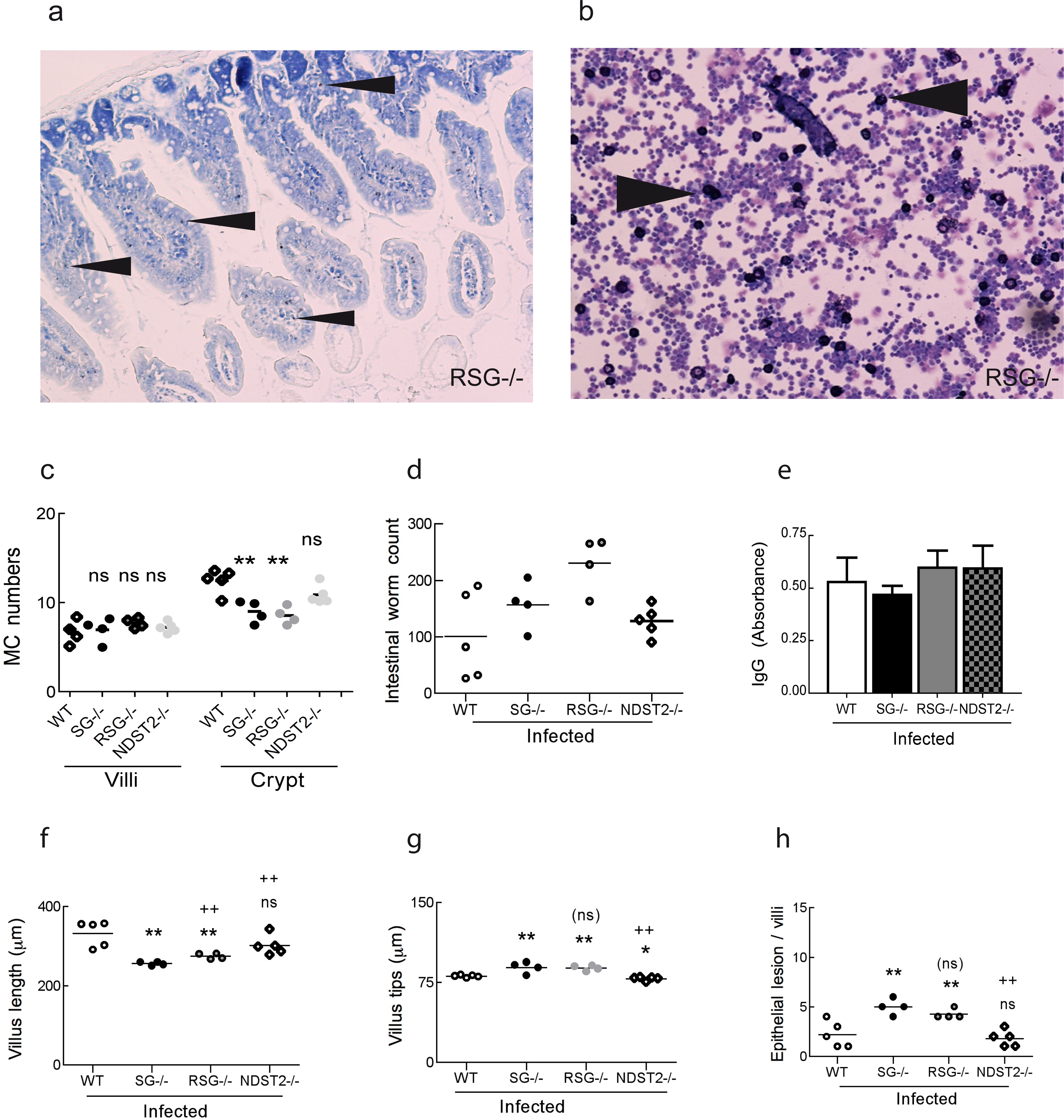

Supplement: Supplementary file 3 — T. spiralis induced entheropathy in serglycin-deficient mice reconstituted with bone marrow derived wild type MCs and in heparin-deficient NDST2−/− mice (12 dpi). The SG−/− mice were reconstituted with 5 x10^6^ WT MCs administered intraperitoneally and 8 weeks later infected with T. spiralis. (a) a representative intestinal section of the infected RSG−/− mice stained with Toluidine blue (pH < 2.0), and (b) a representative cytospin slide of peritoneal cells from an infected RSG−/− mice stained with May-Grünwald/Giemsa. Arrowheads mark the stained MCs. Paraffin embedded intestinal tissues from uninfected and infected WT, SG−/−, MC-reconstituted SG−/− (RSG−/−), and NDST2−/− mice, were sectioned and stained with H&E, and histopathological changes analyzed using Nikon NIS software. (c) MC-counts in chloroacetate esterase stained intestinal tissue, (d) intestinal worm burden, and (e) T. spiralis specific IgG levels in serum. In (f) the villus length, and (g) the swelling of the villi tips was measured of 15 villi per intestinal section as described in M&M. In (h) the epithelial lesion of the villus tip (as defined in Material and Methods) was counted in 10 intact villi per infected mouse. Data from one experiment is shown, with infected mice (N = 4 for RSG−/− and SG−/−, N = 5 for WT and NDST2−/−) and with control mice (N = 3). Data is expressed as mean values (c, d, f-h) and mean + SEM (e), and significant differences between genotypes are indicated in the figure. Not significant, ns P >0.05, *P ≤0.05, **P <0.001, versus infected WT, and not significant (ns) P >0.05, ++P <0.001 versus infected SG−/− mice, respectively. (TIF 9451 kb) [file 12865_2016_155_MOESM3_ESM.tif]

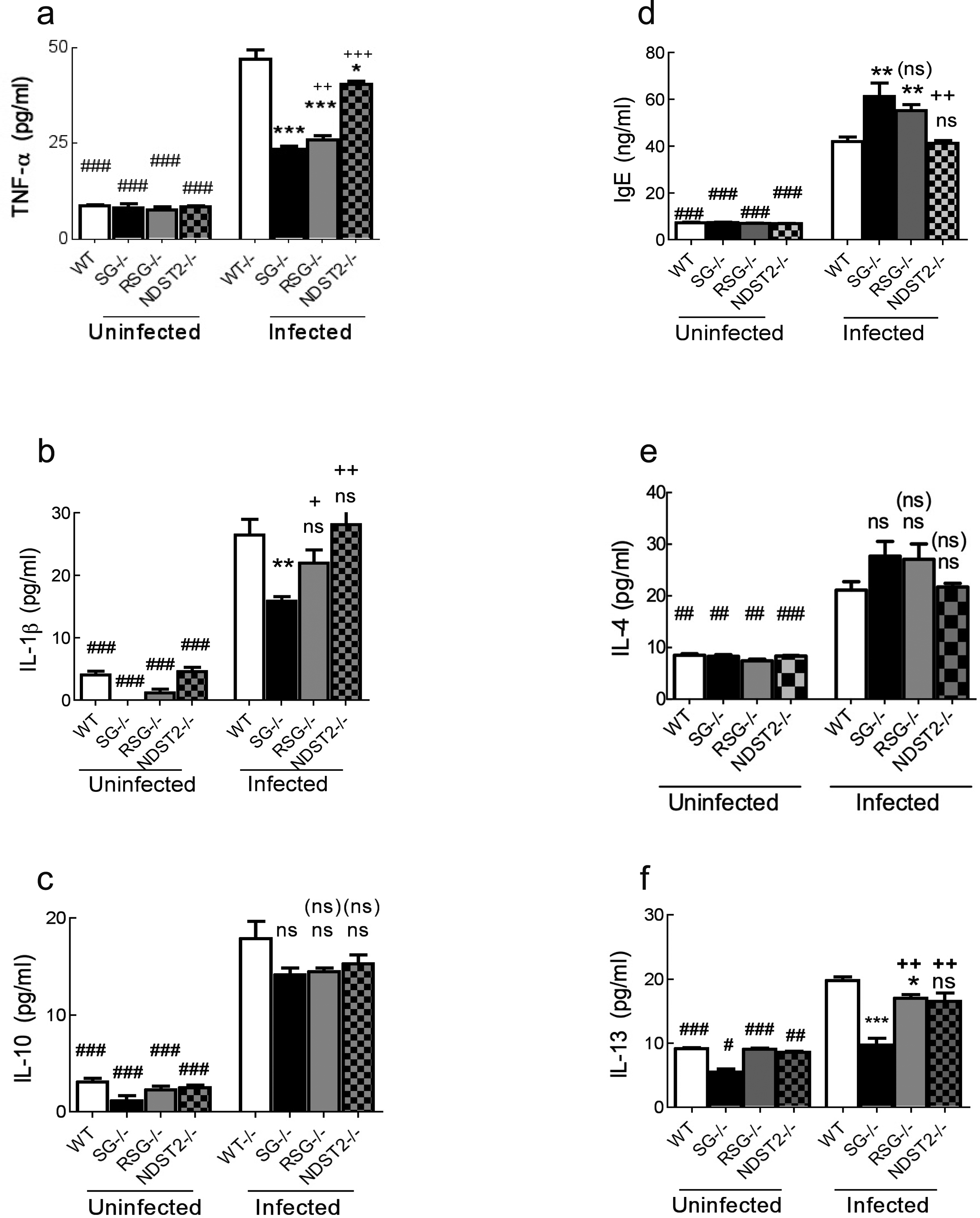

Supplement: Supplementary file 4 — T. spiralis induced cytokine and Th2 profile in heparin-deficient NDST2−/− mice and SG-deficient mice reconstituted with bone marrow derived WT MCs (12 dpi). The levels in serum of TNF-α (a), IL-1β (b), and IL-10 (c), total IgE (d), IL-4 (e), and IL-13 (f) from uninfected and infected WT, SG−/−, MC-reconstituted SG−/− (RSG−/−), and NDST2−/− mice were analyzed by ELISA. Data from one experiment is shown, with infected mice (N = 4 for RSG−/− and SG−/−, or N = 5 for WT and NDST2−/−) and with control mice (N = 3). A statistical significant difference in the levels of (c) IL-10 (WT vs SG−/−, p-value = 0,1294) and (e) IL-4 (WT vs SG−/−, p-value = 0,0758) was not reached in this experiment due to the low number of animals (compare with Fig. 3c and 3e). Data is expressed as mean + SEM and significant differences are indicated in the figure. #P ≤0.05, ##P <0.001, ###P <0.0001 versus infected mice. Not significant, ns P >0.05, *P ≤0.05, **P <0.001, ***P <0.0001 versus infected WT mice, and not significant (ns) P >0.05, +P ≤0.05, ++ P <0.001 versus infected SG−/− mice, respectively. (TIF 1162 kb) [file 12865_2016_155_MOESM4_ESM.tif]

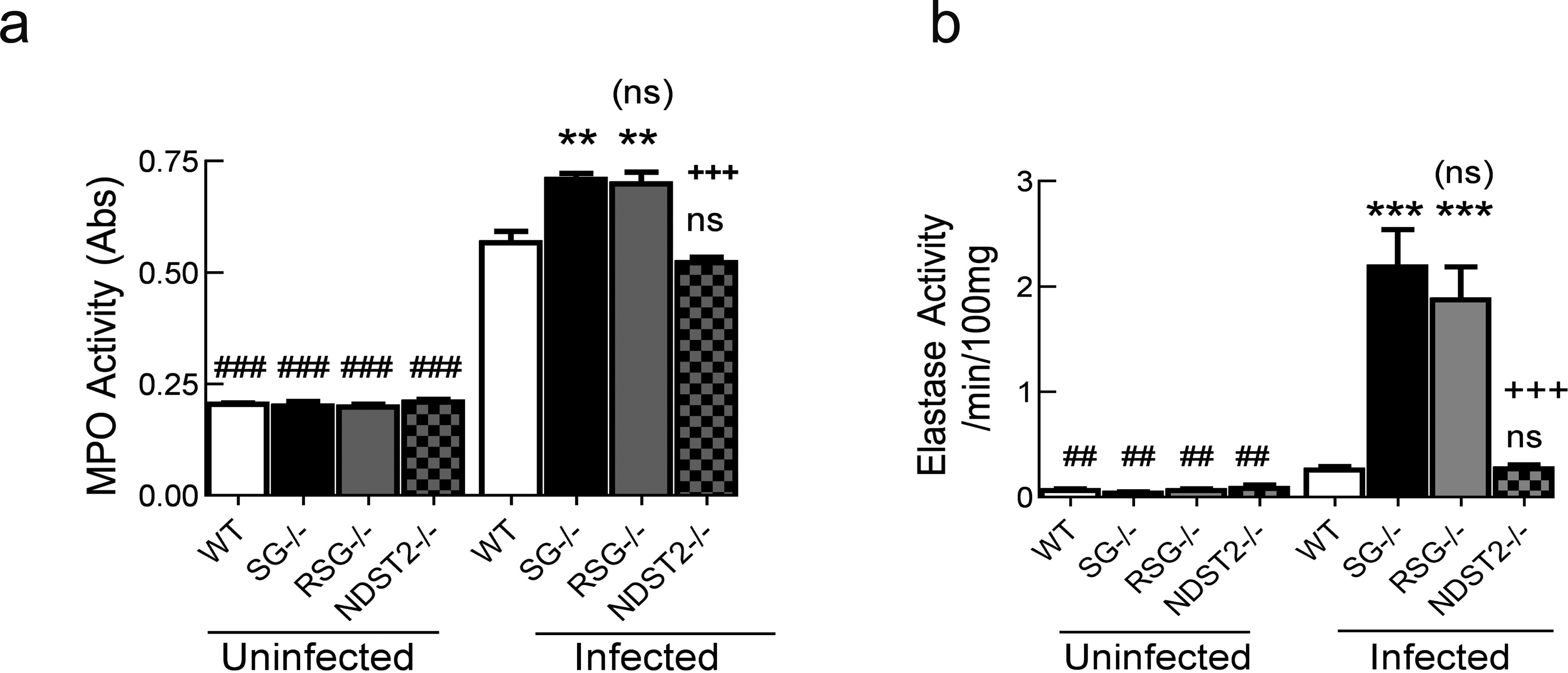

Supplement: Supplementary file 5 — Reconstitution with WT bone marrow derived MCs in serglycin-deficient mice does not dampen the enhanced neutrophil activity (at 12 dpi). Serglycin proteoglycans seem to influence neutrophil and eosinophil activities in T. spiralis infected mice (see Fig. 4 in the main text), and to assess neutrophil and eosinophil recruitment in intestinal tissue in infected serglycin-deficient mice repaired with WT MCs, we measured the enzymatic activities of (a) myeloperoxidase (MPO) and (b) neutrophil elastase (NE). In a and b data from one reconstitution experiment is shown with control mice (N = 3) and with infected mice (N = 4 for RSG−/− and SG−/−, N = 5 for WT and NDST2−/−). Data is expressed as mean + SEM and significant differences are indicated in the figure. ##P <0.001, ###P <0.0001 versus infected mice. Not significant, ns P >0.05, **P <0.001, ***P <0.0001 versus infected WT mice, and not significant (ns) P >0.05, +++P <0.0001 versus infected SG−/− mice, respectively. (TIF 397 kb) [file 12865_2016_155_MOESM5_ESM.tif]
